# Neural Correlates of Aging-Related Differences in Pro-active Control in a Dual Task

**DOI:** 10.3389/fnagi.2021.682499

**Published:** 2021-09-30

**Authors:** Juliana Yordanova, Patrick D. Gajewski, Stephan Getzmann, Roumen Kirov, Michael Falkenstein, Vasil Kolev

**Affiliations:** ^1^Institute of Neurobiology, Bulgarian Academy of Sciences, Sofia, Bulgaria; ^2^Leibniz Research Centre for Working Environment and Human Factors, Dortmund, Germany; ^3^Institute for Working, Learning and Aging, Bochum, Germany

**Keywords:** dual task, proactive control, aging, psychological refractory period (PRP) paradigm, EEG, ERP, slow cortical potentials

## Abstract

**Background:** Multi-tasking is usually impaired in older people. In multi-tasking, a fixed order of sub-tasks can improve performance by promoting a time-structured preparation of sub-tasks. How proactive control prioritizes the pre-activation or inhibition of complex tasks in older people has received no sufficient clarification so far.

**Objective:** To explore the effects of aging on neural proactive control mechanisms in a dual task.

**Methodology:** To address this question, the psychological refractory period (PRP) paradigm was used. Two 2-alternative-forced-choice reaction tasks with a predefined order (T1 and T2) signaled by a cue had to be executed simultaneously or consecutively by young (mean age 25.1 years, *n* = 36) and old subjects (mean age 70.4 years, *n* = 118). Performance indices of dual-task preparation were used to assess the focused preparation of T1 and T2. To compare preparatory mechanisms at the neurophysiologic level, multi-channel electroencephalogram (EEG) was recorded and negative slow cortical potentials (SCPs) were analyzed as objective markers of the amount and localization of cortical pre-activation before sub-task presentation.

**Results:** Dual-task performance was significantly slower in old adults. T1 performance was facilitated in both age groups, but T2 processing in old adults was not optimized by the temporal structure as efficiently as in young adults. Also, only young adults manifested a stable pattern of focused of negative slow-wave activity increase at medial frontal and right-hemisphere posterior regions, which was associated with a coordinated preparatory T1 pre-activation and T2 deferment, while old adults manifested a broad topographic distribution of negative SCPs associated with a pre-activation of sensory and motor processes.

**Conclusions:** These observations demonstrate that the proactive preparation for dual tasking is altered with aging. It is suggested that in young adults, attention-based pre-activation of working memory and inhibitory networks in the right hemisphere synchronizes the simultaneous preparation of the two sub-tasks, whereas in old adults, sensory and motor networks appear to be non-specifically pre-activated for subsequent deferred mode of processing.

## Introduction

Typical everyday tasks consist of a sequence of multiple sub-tasks. Although multi-tasking has as a rule detrimental effects on behavior (e.g., May and Elder, 2018), there is evidence that the temporal organization of sub-tasks in a goal-directed order is a key element of successful performance. Specifically, a constant order of sub-tasks improves performance relative to conditions, in which the order of sub-tasks varies (Luria and Meiran, 2003, 2006), indicating that the fixed temporal structure promotes behavioral efficiency.

**FIGURE 1:**
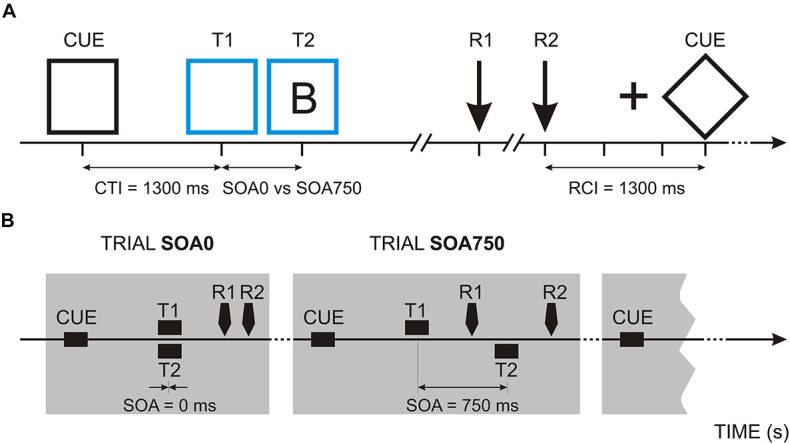
Psychological refractory period (PRP) task: **(A)** Procedure of a single experimental trial. In the example, a blue frame and the letter B are presented as target stimuli. CTI, Cue-target interval; SOA, stimulus onset asynchrony; T1/R1, first target and reaction time in response to the first target; T2/R2, second target and reaction time in response to the second target; RCI, response-cue interval. **(B)** Trials with SOA = 0 ms and SOA = 750 ms. Response times are provisory.

The benefit from keeping the task order fixed can be studied in multi- or dual-task conditions where single sub-tasks have a predefined temporal structure. One such established dual-task condition is the psychological refractory period (PRP) paradigm (Figure 1A). In the PRP paradigm, participants execute two sub-tasks (T1 to be performed first and T2 to be performed second) which are separated by different temporal intervals (e.g., Pashler and Johnston, 1989; De Jong, 1995; Luria and Meiran, 2003). So far, the PRP paradigm has been mainly employed to study the sources of dual-task costs due to sub-task overlap (Pashler and Johnston, 1989). Despite indications that top-down cognitive control networks subserving both proactive and reactive adaptive strategies (Braver, 2012) are critically involved in dual-task costs (e.g., Pashler and Johnston, 1989; Logan and Gordon, 2001; Tombu and Jolicśur, 2003; Worringer et al., 2019, for review), the specific role of proactive neurocognitive mechanisms in preparing dual-task performance has only recently been focused on (Steinhauser and Steinhauser, 2018). Addressing this question is of particular relevance for conditions with altered control or compromised cognitive reserve such as aging (Karayanidis et al., 2011; Robertson, 2014; Gajewski et al., 2020a).

It is well documented that the ability of older individuals for dual tasking is diminished (Allen et al., 1998; Glass et al., 2000; Verhaeghen and Cerella, 2002; Verhaeghen et al., 2003; Maquestiaux et al., 2004; Thönes et al., 2018). Also, modes of cognitive control change with increasing age even in single sensorimotor tasks (Gajewski et al., 2018a,b). Previous studies indicate that aged individuals may not fully implement task settings before target onset, after which they need to over-activate reactive control systems to compensate for the omitted preparation, thus manifesting neural under-recruitment for proactive and an over-recruitment for reactive cognitive control (Hu et al., 2012; Kopp et al., 2014; Van Gerven et al., 2016, 2017). However, whether and how task order set (TOS) supports the preparation for dual-task performance in older people has received no sufficient clarification so far. Therefore, the main objective of the present study was to evaluate the effects of aging on pro-active preparation for dual-task performance.

For that aim, we used a version of the PRP paradigm with a predefined (fixed) order of simultaneously or consecutively presented visuo-motor sub-tasks (Thönes et al., 2018). One sub-task was a choice-reaction letter discrimination task, while the other sub-task was a choice-reaction color discrimination task. The order of sub-tasks was fixed in separate blocks. Although a cue was delivered to indicate which of the two sub-tasks needed to be performed first, it only served to enable a temporal preparation for the dual-task.

Second, in the present study, specific performance indices of dual-task preparation were introduced. Existing behavioral models posit that dual-task preparation can be organized either as a preparation limited only to sub-task T1, or as separate preparatory sets for the two consecutive sub-tasks, or as one superordinate dual-task set (De Jong, 1995; Meyer and Kieras, 1997; Luria and Meiran, 2003). A study by Steinhauser and Steinhauser (2018) has provided original neurophysiologic evidence for a multi-level mode of dual-task preparation that not only activates T1, but also prepares a deferred mode for a fast and efficient T2 processing. With this regard, the performance indices of dual-task preparation used here aimed to assess the focused preparation of the first and the second sub-task in the temporally structured dual-task set.

Third, in the present study, to compare pro-active preparatory mechanisms at the neurophysiologic level, slow cortical potentials (SCPs) were analyzed as objective markers of cortical pre-activation (Khader et al., 2008; Brunia et al., 2012). In paired-stimulus conditions, a fronto-central negative slow potential with midline maximum known as the contingent negative variation (CNV) has originally been described before task stimuli (Walter et al., 1964). The CNV is recognized as a correlate of anticipatory attention toward a target (Walter et al., 1964; Tecce, 1972) as being enhanced when participants are instructed to increase effort on certain trials (e.g., Falkenstein et al., 2003; De Loof et al., 2019). Target-preceding negativities with broader topographic distribution have also been found immediately preceding task stimulus onset (Brunia, 1988). Specific topographic aspects of these negative SCPs are originally described as stimulus-preceding negativities and response-preceding negativities (van Boxtel and Brunia, 1994; Brunia and van Boxtel, 2004; Brunia et al., 2012). According to the threshold-regulation model of slow-wave generation (Elbert, 1993), substantiated by combined EEG-fMRI studies (Hinterberger et al., 2003; Schicke et al., 2006; Khader et al., 2008), negative SCPs represent a measure of the excitability of task-relevant cortical neuronal networks. Accordingly, the topography and amplitude of negative SCPs have been found to be strongly task-dependent reflecting the targeted pre-activations of sensory and motor regions as well as associative regions supporting the implementation of executive proactive processes (e.g., Khader et al., 2008; Brunia et al., 2012; Liebrand et al., 2017; Berchicci et al., 2020).

With this regard, in the present study, the magnitude of negative SCPs was analyzed to assess the amount of pre-activation, and topographic distribution was analyzed to reflect regional patterns of pre-activation, which would correspond to a preparatory involvement of specific cortical regions. It was hypothesized that in case of efficient TOS functioning for proactive cortical pre-activation, the task-to-be-responded first would induce region-specific preparatory patterns involving a fronto-medial CNV. With regard to the predominant involvement of the left hemisphere in verbal processing (Majerus et al., 2006, 2010) and of the right hemisphere in color discrimination (Sasaki et al., 2007; Barnett, 2008), it was also expected that negative SCPs would be lateralized to the left hemisphere for letter-first and to the right hemisphere for color-first sub-task (Rösler et al., 1995b,a; Brunia and van Boxtel, 2004). Further, according to the observations of Steinhauser and Steinhauser (2018), enhanced frontal negative potentials in dual-task conditions are linked to a neural mechanism that defers the implementation of the task set of T2 until T1 execution is completed and represent an inhibition process that suppresses response sets that are conflicting with the immediate task (T1). It was therefore also hypothesized that an efficient proactive deferment (inhibition) of T2 would correspond to enhanced negative SCPs at frontal regions (Steinhauser and Steinhauser, 2018). Finally, the implementation of cognitive control and associated pre-activation of attention and memory networks was expected to induce bilateral fronto-parietal slow-wave patterns in the cue-S1 interval (Worringer et al., 2019).

In sum, in the present study, performance parameters of dual-task preparation and SCPs were compared between young and old adults to evaluate the effects of aging on pro-active preparation for dual-task performance.

## Materials and Methods

### Participants

Participants were young and old subjects recruited through newspaper advertisements and flyers distributed in the city of Dortmund (Germany). All data were gathered as part of a larger training study with a pre- and a post-measure (for details, see Gajewski and Falkenstein, 2012; Gajewski et al., 2020b), with only the pre-measure data being reported here. The samples of young and old subjects analyzed here were taken for re-analysis from the data set reported by Thönes et al. (2018). The group of old subjects included 118 participants (mean age = 70.4 years, SD = 4.2, range = 63–88 years; 75 female), and the sample of young subjects included 36 healthy volunteers (mean age = 25.2 years, SD = 2.7, range = 19–33 years, 19 female). Mini Mental State Examination was conducted to control for the inclusion of only subjects with preserved neurocognitive abilities (Folstein et al., 1975). Additionally, a battery of neuropsychological tests was included to measure attentional endurance, speed of processing and vigilance (Digit-Symbol-Test), short-term and working memory (Digit-Span-Test) as subtests of the Wechsler Adult Intelligence Scale (WAIS-III), interference (Stroop color-word test), Verbal Learning and Memory Test, and psychomotor speed of task switching (Trail Making Test), see Supplementary Table 1. Neuropsychological tests were administered in an extra session, one day before the EEG session. They demonstrated the expected aging-related difference in neurocognitive functions (Supplementary Table 1; Gajewski et al., 2018a; Thönes et al., 2018). All participants had normal or corrected-to-normal vision and hearing, and gave informed written consent according to the Declaration of Helsinki. They received financial compensation or partial course credit (for the students) for their participation. The whole study was approved by Ethic Committee of the Leibniz Research Centre for Working Environment and Human Factors, Dortmund. The participants provided their written informed consent to participate in this study.

### Stimuli and Task Procedure

As described in Thönes et al. (2018), all stimuli were presented centrally on a computer screen (size: 17″, refresh rate: 100 Hz, resolution: 640 × 480 pixels) at a viewing distance of 60 cm. A fixation point (5 mm × 5 mm) was presented before each stimulus and located in the center of the monitor. Responses to each target stimulus were given by pressing one of four designated keys on a response box.

Stimuli are illustrated in Figure 1A. On each trial, a centrally presented diamond or a square (37 mm per side) with a duration of 1,300 ms served as a cue stimulus. The diamond was used as a cue for the task order letter-color and the square—for the task order color-letter. Target stimuli were either a white square or a diamond frame (corresponding to the cue) that changed the color from the black cue into blue or yellow (color discrimination task) and a letter, B or D (letter discrimination task), presented within the frame. Randomly (*p* = 0.5), the targets S1 and S2 were either presented simultaneously (0 ms stimulus onset asynchrony; SOA = 0 ms) or S2 appeared 750 ms after S1 (SOA = 750 ms).

Sub-tasks: The two sub-tasks were (1) to discriminate blue from yellow frames, and (2) to discriminate letter D from letter B—Figure 1A. On a response box, four response keys were set. In the upper row of the response box, the responses for the color choice were given. In the lower row, the responses for the letter choice were given. According to the instructions, participants had to respond to the blue frame by pressing the button with the left index finger, to the yellow frame by pressing the button with the right index finger, to the letter B by pressing the left middle-finger button, and to the letter D by pressing the right-middle finger button. The stimulus-response mapping was the same for all participants. Thus, the subjects performed two 2-alternative-forced-choice reaction tasks.

Trial structure is illustrated in Figure 1B. Each cue was followed either by two target stimuli (S1 and S2) presented simultaneously (SOA = 0) or in a rapid succession (SOA = 750 ms). In the first block, the first sub-task was to discriminate letters, and the second sub-task was to discriminate colors (B1-LC). In the second block, where the order of sub-tasks was reversed, the subject had to respond to the frame color first and then to the letter (B2-CL). The subjects were instructed to respond to the target stimuli as quickly and as accurately as possible in the defined order, i.e., with priority to T1 response (R1). Both stimuli remained until the second response (R2) was completed. After R2 had been given, a blank screen was presented for 500 ms, followed by a fixation cross for 500 ms and a blank screen again for 300 ms, which indicated the beginning of the next trial. Thus, as indicated on Figure 1A, the interval between R2 and the cue of the next trial was always 1,300 ms. In total, 68 trials were presented to each participant. Prior to the experiment, participants were instructed to practice the dual task until they felt comfortable with it.

### Electroencephalogram Recording and Processing

The electroencephalogram (EEG) was recorded continuously from 32 active BioSemi pin-type electrodes arranged according to the extended 10–20 system and mounted on an elastic preconfigured cap (Easycap GmbH, Germany). The montage included 8 midline sites and 12 sites on each hemisphere and two mastoid electrodes as reference. The horizontal and vertical electro-oculogram (EOG) was recorded bipolarly from electrodes at both eyes. Eye movement artifacts were corrected by using the correction algorithm of Gratton et al. (1983). Electrode impedances were kept below 10 kΩ. EEG was amplified with cut-off frequencies 0.01 and 140 Hz. EEG and EOG were sampled with a rate of 2,048 Hz. Offline, the EEG was downscaled to a sampling rate of 1,024 Hz and segmented into cue-locked epochs with a duration 1,800 ms and a baseline of 300 ms before the cue. Epochs in which the amplitude exceeded ±150 μV were rejected. Analyses were performed using Brain Vision Analyzer ver. 2.2.1 (Brain Products GmbH, Germany). For analysis, the EEG epochs were filtered digitally with a band-pass 0.03 and 12 Hz, and CSD-transformed to enhance the spatial resolution of the analyzed signals and support inferences about sources of their generation (Perrin et al., 1989, 1990).

### Analysis Parameters

#### Performance

Reaction times (RT) to targets in T1 and T2 sub-tasks in SOA = 750 ms (SOA750) and SOA = 0 ms (SOA0) conditions were recorded and analyzed along with commission and omission errors. To assess dual-task preparation, performance data were analyzed with respect to (1) the speed of R1 to reflect the behavioral correlate of T1 preparation, (2) the speed of R2 relative to R1 in the SOA750 condition to reflect the promoting/inhibiting effect of TOS on T2 execution, and (3) the speed of R1 in the SOA0 and SOA750 conditions to reflect the effect of input interference. In addition, to refine the comparison of preparation-related processes between age groups, the following normalized indices of focused preparation for T1, T2, and interference control were introduced. The computation algorithms are presented in Table 1.

**TABLE 1:** RT parameters and calculation.

**Parameter**	**Computation**
Capacity (ms)	*GC* = (*B*_1_*RT*_1_SOA_750_ + *B*_1_*RT*_1_SOA_0_ + *B*_1_*RT*_2_SOA_750_ + *B*_1_*RT*_2_SOA_0_ + *B*_2_*RT*_1_SOA_750_ + *B*_2_*RT*_1_SOA_0_ + *B*_2_*RT*_2_/SOA_750_ + *B*_2_*RT*_2_SOA_0_)/8
Preparation of T1 (*PT*1) (%)	$P\,T\,1=\frac{\frac{R\,T\,1\,{\text{SOA}}_{0}+R\,T\,1\,{\text{SOA}}_{750}}{2}}{G\,C}*100-100$
Preparation of T2 (*PT*2) (%)	$P\,T\,2=\frac{R\,T\,2\,{\text{SOA}}_{750}}{R\,T\,1\,{\text{SOA}}_{750}}*100-100$
Input Interference (*IIT*1) (%)	$I\,I\,T\,1=\frac{R\,T\,1\,{\text{SOA}}_{750}}{R\,T\,1\,{\text{SOA}}_{0}}*100-100$

*B_*x*_*RT*_*x*_SOA_*y*_, reaction time, where *B*_*x*_ – block (*x* = 1 or 2), *RT*_*x*_ – reaction time of respective target (*x* = 1 or 2), SOA_*y*_ – stimulus onset asynchrony (*y* = 0 or 750 ms); *RT*1SOA_*x*_, reaction time to target 1, where *RT*1SOA_*x*_ = (*B*1*RT*1 + *B*2*RT*1)/2, *x* = 0 or 750; *RT*2SOA_*x*_, reaction time to target 2, where *RT*2SOA_*x*_ = (*B*1*RT*2 + *B*2*RT*2)/2, *x* = 0 or 750.*

1. Capacity, defined as the overall speed of information processing in the dual-task blocks as reflected by the mean RT in T1 and T2 in two SOA conditions (SOA750 and SOA0) and two blocks (B1-LC and B2-CL).
2. Preparation of T1 (PT1), defined as the relative speeding of T1 execution as contrasted to the overall capacity. The computed PT1 value captures the rate of RT1 decrease/increase relative to mean RT (Capacity). Small values reflect a facilitated T1 execution and a focused T1 preparation.
3. Preparation of T2 (PT2), defined as the relative speeding/slowing of T2 execution as contrasted to T1 in the SOA750 condition. Small values reflect a facilitated T2 execution and a strong preparation for T2 execution.
4. Input Interference (IIT1), defined as the normalized difference between RT1 in the SOA750 and SOA0 conditions. The measure reflects the rate of change of RT1 during simultaneous relative to single T1 processing. Small values reflect a facilitating effect of sub-task overlapping and large values are regarded as indicating a delaying effect of input interference.

RT1 and RT2 as well as error rates were compared between the two age groups using analysis of variance (ANOVA) with one between-subjects variable *Age* (young vs. old) and within-subjects variables *Block* (B1 vs. B2) and *Task* (T1 vs. T2)/*SOA* (SOA750 vs. SOA0), depending on the design. Dual-task performance parameters (Capacity, PT1, PT2, and IIT1) were analyzed by *Age x Block* ANOVAs.

#### Event-Related Potentials and Slow Negative Potentials

All analyses and visualizations were performed using Brain Vision Analyzer, ver. 2.2 (Brain Products GmbH, Germany). SCPs were measured as the mean value of the activity 300 ms before the first target (S1), with 300 ms pre-cue baseline, from the electrodes F3/Fz/F4, FC3/FCz/FC4, C3/Cz/C4, CP3/CPz/CP4, P3/Pz/P4, PO3/POz/PO4, and O1/Oz/O2. Slow-wave magnitude was subjected to a repeated-measures ANOVA with a between-subjects variable *Age* (Young vs. Old) and within-subjects variables *Block* (B1-LC vs. B2-CL) x *Laterality* (Left vs. Midline vs. Right) x *Region* (7 levels corresponding to frontal, fronto-central, central, parieto-central, parietal, parieto-occipital and occipital regions), with SOA0 and SOA750 trials being collapsed. The objectives of these analyses were to assess condition and age differences in the amount of preparatory activation (*sufficiency* of activation), and the *efficiency* of preparatory activation in terms of pre-activation of task-specific cortical regions.

To test the associations between local negative potentials and specific aspects of dual-task preparation, stepwise regression models were computed. In each model, the dependent variable was one of the parameters reflecting the preparation of T1 (PT1), T2 (PT2), or input interference control (IIT1), and predictors were SCPs at 21 single electrodes. In addition, since the parameters of dual-task preparation (PT1, PT2, and IIT1) were inter-correlated (see Supplementary Table 2), Capacity and the remaining preparation parameters were included as predictors to control for the internal correlations between preparation parameters and to guarantee that any extracted SCP predictor was independent and did not originate from correlations with the other preparation parameters. Because performance results implied aging-dependent differences in dual-task preparation strategies, regression analyses were conducted separately for each age group. All analyzed parameters manifested a normal distribution across and within age groups as indicated by the Kolmogorov–Smirnov test (0.127 < *p* < 0.991).

## Results

### Performance

**FIGURE 2:**
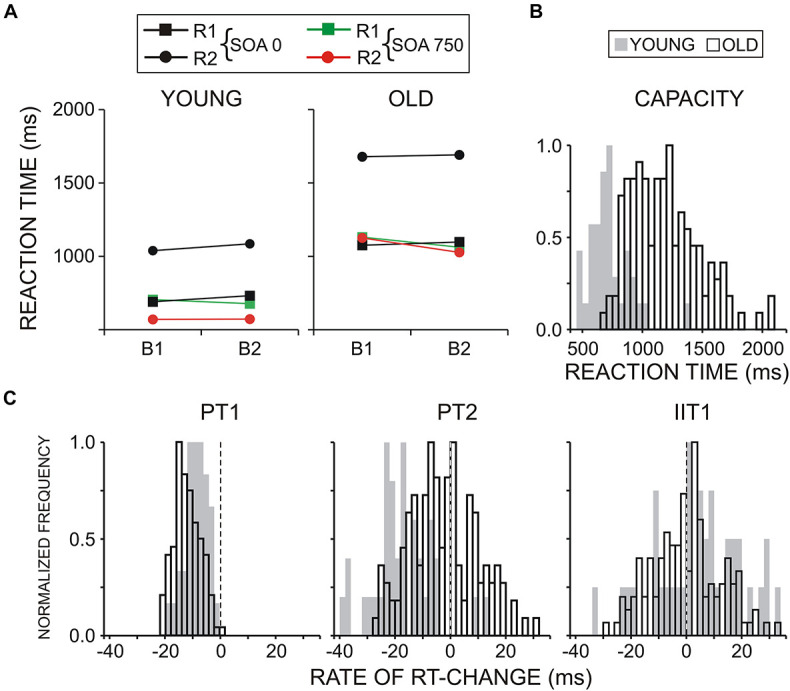
**(A)** Reaction time (RT) for two groups of participants, young and old. **(B)** Normalized distribution of Capacity parameter for both groups of young and old adults. Capacity: speed of processing in the dual-task blocks as reflected by the mean RT to S1 and S2 in two SOA conditions (SOA750 and SOA0), in two blocks (B1-LC and B2-CL). **(C)** Normalized distribution of dual-task preparation parameters: preparation for T1 (PT1), preparation for T2 (PT2), and input interference for T1 (IIT1), presented for young and old subjects.

Figure 2 demonstrates that RT (collapsed across B1 and B2, and SOA0 and SOA750 conditions) was overall substantially slower in old than young adults [*Age*, *F*(1/152) = 81.6, *p* < 0.0001, η_*p*_^2^ = 0.331; group mean RT ± SD of young participants = 778 ± 195 ms; group mean RT ± SD of old participants = 1,239 ± 325 ms], which referred to both RT1 and RT2 (group mean RT1 ± SD = 720 ± 210 ms vs. 1,090 ± 305 ms; RT2 ± SD = 835 ± 184 ms vs. 1,385 ± 343 ms). The delay of RT2 relative to RT1 was larger in old (mean 550 ± 128 ms) than young (mean 370 ± 75 ms) adults [*Age* × *Task*, *F*(1/152) = 59.5, *p* < 0.0001; η_*p*_^2^ = 0.265].

Also, there was a significantly larger delay of RT2 in the SOA0 condition in old (mean 602 ± 152 ms) as compared to young (mean 353 ± 92 ms) adults [*Age* × *Task* × *SOA F*(1/152) = 58.1, *p* < 0.0001; η_*p*_^2^ = 0.261]—Figure 2A. No significant main or interactive effects of *Block* were yielded.

Analysis of dual-task preparation indices detailed the observations from RT analysis. As shown in Figure 2C, group mean negative values of PT1 revealed a relative speeding of responses to T1 in the two age groups, implying a focused preparation of T1 that was also more prominent in old adults [*Age*, *F*(1/152) = 19.9, *p* < 0.0001, η_*p*_^2^ = 0.108]. Notably, in the two age groups, R2 responses were faster than R1 responses in the SOA750 condition as indexed by negative PT2 values (Figure 2C), although R2 speeding was significantly more expressed in young subjects [*Age*, *F*(1/152) = 41.2, *p* < 0.0001, η_*p*_^2^ = 0.202] due to R2 speeding in only about half of old participants (χ^2^ = 22.8, *p* < 0.001). The positive values of IIT1 demonstrate the presence of input interference (i.e., a RT1 delay in the simultaneous SOA0 relative to the consecutive SOA750 condition). Yet, aging did not affect significantly the amount of input interference [*Age*, *F*(1/152) = 3.4, *p* = 0.07, η_*p*_^2^ = 0.02].

No difference in accuracy existed between the groups of young and old adults [*Age*, *F*(1/152) = 0.08, *p* = 0.77]. Error rate was higher in the SOA750 condition [*SOA*, *F*(1/152) = 4.01, *p* = 0.05, η_*p*_^2^ = 0.03], which was observed in the two groups (*Age* × *SOA*, *p* > 0.2).

### Effects of Aging on Slow Negative Potentials

**FIGURE 3:**
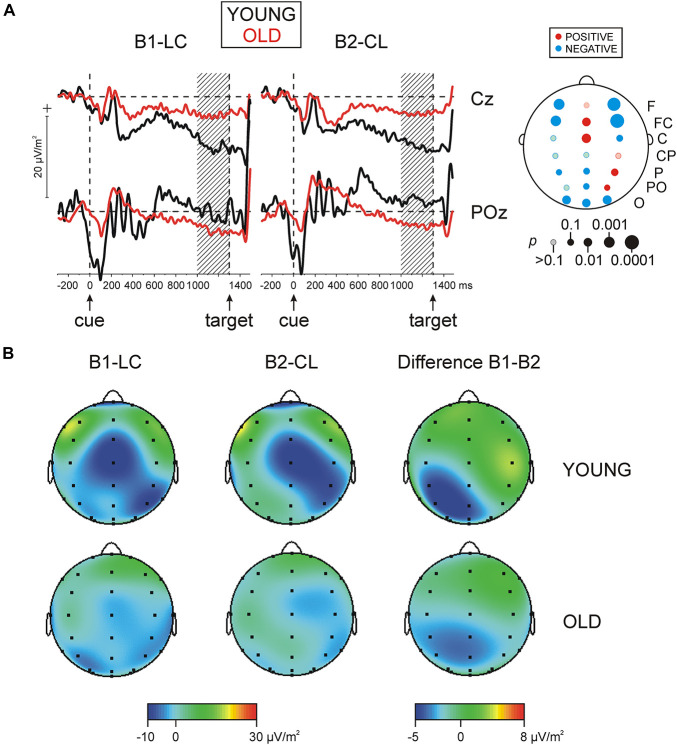
**(A)** Grand average of slow cortical potentials (SCPs) for two groups, young and old, for two blocks, block 1 (B1-LC), and block 2 (B2-CL) at electrodes Cz and POz (band pass 0.03-30 Hz). Statistical significance of difference in SCPs between groups of young and old subjects is presented in the map on the right side. *p*, statistical significance; red (negative SCPs young > negative SCPs old), blue (negative SCPs old > negative SCPs young). **(B)** Grand average maps of the distribution of SCPs for the same two blocks and for the B1–B2 difference in young and old adults.

The main effect of *Age* on SCP magnitude was significant [*F*(1/152) = 21.9, *p* < 0.001, η_*p*_^2^ = 0.126] due to overall more negative SCP values in old relative to young adults. Figures 3A,B demonstrate that the regional distribution of negative SCPs differed between young and old adults, which was supported by the significant interactions *Laterality* × *Age* [*F*(6/912) = 3.9, *p* = 0.05, η_*p*_^2^ = 0.02], and *Region* × *Laterality* × *Age* [*F*(6/912) = 4.2, *p* < 0.001, η_*p*_^2^ = 0.028]. Testing topography effects in each age group revealed that in young subjects, negative SCPs were maximally expressed at FCz and Cz and over the right parietal cortex [*Region* × *Laterality* in young adults, *F*(12/420) = 4.1, *p* = 0.002, η_*p*_^2^ = 0.11]. In contrast, negative SCPs of old adults were mainly expressed at parieto-occipital regions [*Region*, *F*(6/702) = 3.2, *p* = 0.02, η_*p*_^2^ = 0.027; *Region* × *Laterality*, *F*(12/1404) = 1.5, *p* = 0.16, η_*p*_^2^ = 0.013]. Accordingly, as depicted in Figure 3A (upper right), testing simple *Age* effects at each electrode yielded significantly larger (more negative) negative SCPs in young relative to old subjects at FCz, Cz, and P4 [*Age*, *F*(1/152) = 3.8 ÷ 7.8, *p* = 0.0 ÷ 0.006, η_*p*_^2^ = 0.025 ÷ 0.022], and significantly larger negative SCPs in old relative to young adults at occipital electrodes O1, Oz, and O2 [*Age*, *F*(1/152) = 6.2 ÷ 7.4, *p* = 0.01 ÷ 0.007, η_*p*_^2^ = 0.02 ÷ 0.046)—Figure 3A. In addition, at bi-lateral frontal and fronto-central electrodes (F3, FC3, F4, and FC4) slow potentials were overall negative in old adults, whereas they manifested positive values in young adults, yielding significant *Age* effects at these locations [*F*(1/152) = 10.3 ÷ 15.8, *p* = 0.002 ÷ 0.001, η_*p*_^2^ = 0.064 ÷ 0.103].

The main effect of the *Block* factor was not significant. However, a significant *Block* × *Laterality* interaction [*F*(2/304) = 3.9, *p* = 0.02, η_*p*_^2^ = 0.015] reflected an asymmetric distribution of negative SCPs in each block—Figures 3A,B. Specifically, the significant *Block* effect over the left hemisphere [*F*(1/152) = 3.8, *p* = 0.05, η_*p*_^2^ = 0.025] stemmed from larger negative SCPs for the letter-first (B1-LC) task, specifically focused to CP3, P3, and PO3 locations [*Block* × *Region*, *F*(6/912) = 2.5, *p* = 0.05, η_*p*_^2^ = 0.016; *Block* effects at these electrodes, *F*(1/152) = 3.7 ÷ 5.8, *p* = 0.05 ÷ 0.017]. In contrast, the significant *Block* effect over the right hemisphere [*F*(1/152) = 6.8, *p* = 0.01, η_*p*_^2^ = 0.019] stemmed from larger negative SCPs for the color-first (B2-CL) as compared to the letter-first (B1-LC) task. Importantly, this effect was detected in the two age groups (*Block* × *Age* effects, *p* = 0.3 ÷ 0.9) indicating a greater pre-activation of the right hemisphere during preparation for the color discrimination task, and a greater pre-activation of the left hemisphere during preparation for the letter discrimination task, independently of age.

### Associations Between Slow Negative Potentials and Dual-Task Preparation

The detailed statistical description presented in the Supplementary Table 3 shows that for each dual-task preparation parameter, one or more preparation parameters were extracted as independent predictors. However, negative SCPs at specific locations also were extracted as predictors indicating their independent roles for dual-task preparation.

In the group of young adults, larger negative potentials (corresponding to smaller SCP values) at Cz, P4 and CP3 predicted stronger T1 preparation (smaller PT1 values, *p* = 0.04 ÷ 0.001), whereas larger negative potentials at C4 and O2 predicted weaker T1 preparation (larger PT1 values, *p* = 0.007 ÷ 0.001). Likewise, larger negative potentials at right-hemisphere electrodes F4, C4 and P4 predicted weaker T2 preparation or delayed T2 execution reflected by larger PT2 (*p* = 0.01 ÷ 0.005), while enhanced negativity at FCz was positively associated with T2 preparation (*p* = 0.02). For IIT1, however, negative wave increase at C4 predicted improved interference control as reflected by IIT1 decrease (*p* = 0.03).

In the group of old adults, larger negative potentials at Fz, P3, and PO3 predicted a stronger preparatory focus to T1 (smaller PT1 values, *p* = 0.02 ÷ 0.04), as well as to T2 (smaller PT2 values, *p* = 0.05 ÷ 0.009 at Fz and PO3), whereas larger negative potentials at P3 and PO3 were associated with reduced input interference control (smaller IIT1 values, *p* = 0.05).

## Discussion

The present study was undertaken to evaluate the effects of aging on pro-active preparation for dual-task performance. The PRP paradigm with a predefined order of simultaneous or consecutive visuo-motor sub-tasks was used (Thönes et al., 2018). Performance indices of dual-task preparation were introduced to assess at the behavioral level the focused preparation of the first and the second sub-task in a temporally structured dual-task set. To compare preparatory mechanisms at the neurophysiological level, slow negative potentials were analyzed as objective markers of cortical pre-activation (Khader et al., 2008; Brunia et al., 2012). It was hypothesized that in contrast to single-task preparation whereby preparatory resources can specifically target single-task sensory, motor or cognitive mechanisms, the preparatory activity supporting dual-tasking may require a different organization following the necessity to simultaneously prioritize and/or inhibit sub-tasks, which might also depend on aging.

Major results indicate that the ability to make use of the dual-task temporal structure and to optimize T1 processing was preserved in old adults. Yet, in contrast to young subjects, T2 processing in old adults was not efficiently optimized by T2 temporal prediction provided by sub-task structure. As discussed below, the neurocognitive systems involved in pro-active preparation for dual tasking (pre-activation and inhibition) were altered in aged individuals who employed different processing strategies, possibly in an attempt to compensate for decrements in cognitive control mechanisms.

### Dual-Task Preparation in Young Adults

Analysis of RT and dual-task parameters demonstrated that young adults applied a complex preparatory strategy to meet the requirements of the dual task employed in the present study. First, as indexed by PT1, there was a relative speeding of responses to S1, suggesting a pro-active preparation of T1 for immediate execution. Also, the lack of delay of reactions to S1 when S1 and S2 were presented simultaneously as compared to when they were presented separately further verified the focused preparation of T1, promoting efficient S1 selection and a high level of input interference control implemented by young adults. These observations are in line with existing models of dual-task performance, all of which emphasize on the dominating preparation for T1 (De Jong, 1995; Meyer and Kieras, 1997). Second, PT2 results showed that in case of sub-task separation by 750 ms, reactions to S2 were faster than reactions to S1 (in all but 2 subjects), pointing to the beneficial effect of TOS on T2 execution. Because group mean RT to S1 of young adults was 720 ms, it is plausible that the interval of 750 ms between S1 and S2 was mostly occupied by T1-related operations of stimulus discrimination, response selection and response execution. It is therefore not likely that the beneficial T2 preparation has taken place during T1 execution, suggesting a pre-existing update and preparation for T2 already during the cue-S1 interval. Furthermore, reactions to T1 in the currently used dual-task condition were substantially longer than typically recorded in young subjects in much more complex (4-choice) visuo-motor single tasks (Yordanova et al., 2004; Kolev et al., 2006), further implying that the capacity for T1 preparation might have been reduced by simultaneous preparatory processes supporting the second sub-task T2. The strong negative correlation (*r* = −0.841, *p* < 0.001) between PT1 and PT2 also demonstrates that the preparation of T1 and T2 was regulated in a coordinated manner. These observations are consistent with models of dual-task preparation according to which both T1 and T2 sets are pre-activated in the cue-S1 interval, being supported by update of either separate T1 and T2 representations, or a superordinate T1T2 representation comprising information about the temporal order structure of the dual-task trial as a whole (De Jong, 1995; Meyer and Kieras, 1997; Luria and Meiran, 2003). These behavioral findings in young adults imply that during the cue-S1 interval (1) both T1 and T2 sensorimotor task sets are updated and maintained in working memory, (2) inhibitory mechanisms are activated in parallel to support input interference and braked T2 inhibition for delayed execution (Meyer and Bucci, 2016), (3) attention resources are deployed to promote sensory and motor processes relevant to immediate T1 execution, and (4) selective attention mechanisms are pre-activated for controlling interference between simultaneous S1 and S2 by prioritizing S1 and filtering S2 representations (Worringer et al., 2019).

Analysis of SCPs in young adults revealed a stable topography pattern characterized by a focused negative potential increase at medial frontal and right-hemisphere posterior regions. This pattern was consistently observed in each block, irrespective of whether the letter or color discrimination task had to be executed first, pointing to the association of negative SCP topography with the employment of general preparatory strategies rather than with a specific preparation for the immediate sub-task. Indeed, between-block comparisons revealed that the altered sub-task order in the two blocks was only associated with a lateralized enhancement of negative potentials depending on whether T1 required letter or color discrimination. Confirming our hypothesis, slow negative potentials were more pronounced over the left hemisphere when the immediate demand was to perform a letter discrimination task as compared to the color-discrimination task (Majerus et al., 2006, 2010) and over the right hemisphere when the immediate task was to discriminate colors as compared to letters (Sasaki et al., 2007; Barnett, 2008). This observation provides a neurophysiologic evidence for the prioritized preparation of T1 processing in terms of increased excitability of cortical regions relevant for upcoming stimulus processing (van Boxtel and Brunia, 1994; Khader et al., 2007; Brunia et al., 2012). Importantly, the asymmetric T1-related negativity increase did not affect the common pattern of predominant negative SCP distribution over the medial frontal and right-hemisphere posterior areas.

**FIGURE 4:**
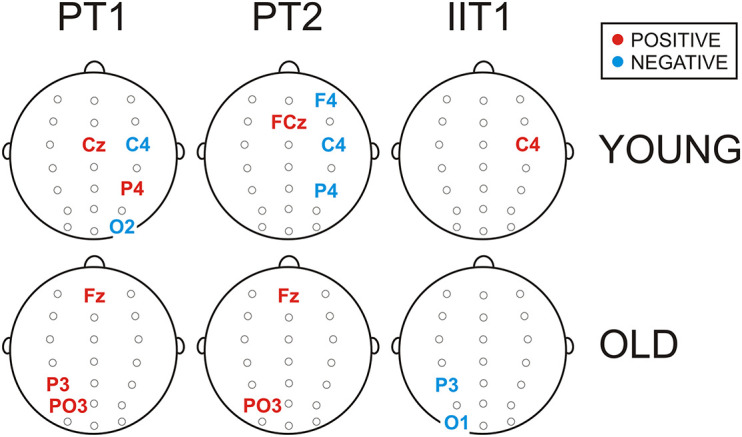
Schematic illustration of electrode positions at which SCPs were extracted by regression models as positive (in red) or negative (in blue) predictors (*p* < 0.05) of T1 preparation (PT1), T2 preparation (PT2), and input interference (IIT1) for the groups of young and old adults. Statistical details are presented in Supplementary Table 3.

In previous single-task studies, the medial frontal slow negative potential identified as CNV (Walter et al., 1964) has been consistently employed as a marker of the amount of cognitive effort devoted to preparation and pro-active task control (e.g., Tecce, 1972; van Boxtel and Brunia, 1994; Chaillou et al., 2018; Gajewski et al., 2020b; Thönes et al., 2018), including proactive inhibitory control (Liebrand et al., 2017; Brevers et al., 2020) and temporal orienting (Berchicci et al., 2020). According to current regression models, enhanced midline fronto-central negative potentials predicted improved performance of both T1 and T2 (Figure 4 and Supplementary Table 3). This observation provides evidence that in dual-task conditions, the CNV is associated with a pro-active facilitation of both the immediate and the deferred sub-task (Steinhauser and Steinhauser, 2018). In addition, basing on the data set analyzed here, Thönes et al. (2018) have demonstrated that enhanced CNV is associated with superior T2 interference control at the response selection stage (i.e., with reduced dual-task costs). Thus, similar to the functional correlations of CNV in single tasks, the stable increase of fronto-medial negative potentials generated in the cue-S1 interval in dual-task conditions reflects the mobilized amount of attention deployed for a variety of cognitive control processes.

A novel observation of the present study was that during dual-task preparation in young adults, there was a pronounced increase of negative potentials at right-hemisphere posterior regions, irrespective of the nature of the immediate sub-task (letter or color). Previously, brain stimulation studies have localized visual working memory mainly in the right hemisphere (Hong et al., 2000; Berryhill and Olson, 2008) and slow negative potentials at parieto-occipital cortical regions have been identified as correlates of maintenance of information in visual working memory (Drew et al., 2006; Barton and Brewer, 2013; Fukuda et al., 2015; Luria et al., 2016). It also has been demonstrated that focusing attention to internal representations in visual working memory is related to activity in the right intraparietal sulcus (Trapp and Lepsien, 2012). More recent research has linked the intraparietal sulcus and anterior cingulate cortex (ACC) as nodes of a fronto-parietal circuit underlying maintenance of visual working memory, whereby ACC activity boosts and protects memory content from decay (Duma et al., 2019). In view of these previous studies and currently found predictive role of the right parietal negativity (P4) for speeded T1 processing (Supplementary Table 3), the enhancement of negative SCP at right parietal regions during dual-task preparation in young adults may be linked to proactive attention-based maintenance of the immediate T1 set in visual working memory.

However, the present results also show that enhanced negative SCPs at right-hemisphere electrodes predicted slowing of both T1 and T2 (Supplementary Table 3). Specifically, right-hemisphere sensory and motor negative SCPs were extracted as negative predictors of PT1, right-hemisphere fronto-centro-parietal negative SCPs were extracted as negative predictors of PT2, and superior input interference control (IIT1) was predicted by SCPs at the right motor cortex—Figure 4. These observations suggest that right-localized slow negative potentials reflect processes of pro-active inhibition of both the immediate and deferred sub-tasks of the dual-task trial. The link between right-lateralized negative waves and pro-active activation of inhibitory networks is further supported by previous research demonstrating the key involvement of the right-hemisphere in fronto-parietal inhibitory networks (Cai et al., 2016; Meyer and Bucci, 2016; Bianco et al., 2017). Likewise, Filmer et al. (2015) have provided evidence for the role of the right parietal cortex for interference control during processing of competing stimuli. With this regard, the currently observed enhancement of negative SCPs at right posterior regions appears to reflect also a common pro-active inhibitory network during dual-task preparation, which controls both immediate and deferred sub-tasks and is not limited to the braked inhibition of the second sub-task (Meyer and Bucci, 2016). Thus, new neurophysiological evidence is provided about co-existent pro-active T1 and T2 inhibition.

### Dual-Task Preparation in Older Adults

Performance analyses confirmed that old adults manifested a substantial slowing in the dual task as compared to young adults (Figures 2A,B; Thönes et al., 2018). Nonetheless, preparation indices PT1, PT2, and IIT1 indicated that similar to young adults, old subjects were able to benefit from a predefined order of sub-tasks by allocating resources to the sub-task-to-be-executed first and by controlling for input interference. However, the beneficial speeding of reactions to the second sub-task was reduced pointing to an inefficient preparation of T2, or delays stemming from over-loaded reactive control (Kopp et al., 2014).

It is notable that despite the benefits of TOS for T1 preparation, responses to T1 were still dramatically slowed down in old subjects (mean group RT1 = 1,090 ms). Electromyographic (EMG) activity reflecting muscle contraction dynamics has been shown to be similar in young and old adults for both simple and binary choice-reaction tasks (Yordanova et al., 2004), implying that overall performance slowing in the older group may not primarily originate from slowing in muscle contraction and motor execution processes. Further, in single-task conditions, older individuals are able to discriminate letters in a four-choice reaction task faster than about 550 ms (Yordanova et al., 2004; Kolev et al., 2006), e.g., almost as twice as fast than in the simpler 2-choice reaction sub-task used here. Hence, it is not plausible that T1 slowing is merely due to inability to produce speeded reactions in the two-choice sub-task T1. T1 execution might be therefore strategically postponed by old subjects to avoid overlapping with S2 delivery in the SOA750 ms condition, in contrast to young individuals who obviously try (or are able) to avoid overlapping by finalizing T1 execution before S2 delivery. Alternatively, a possible bottle-neck for T1 response selection in simultaneous trials might have automatically delayed T1 responses in consecutive trials due to reduced flexibility. Although the lack of RT slowing for the simultaneous as compared to the consecutive sub-task presentation does not infer aging-related deficits in input interference control, such a deficit (Hein and Schubert, 2004) might have remained undetected due to deferred T1 processing. Thus, in contrast to young adults, older adults appear to apply a cognitive strategy of prioritized but deferred first sub-task processing, possibly in an attempt to compensate for overall performance slowing. These results are consistent with the findings of Glass et al. (2000) according to which in dual-task conditions, generalized slowing, process-specific slowing, and the use of more cautious task-coordination strategies in aging lead to increased dual-task costs.

This different preparatory strategy in aged subjects (prioritized but deferred preparation of T1 and inefficient preparation of T2) was accompanied by an altered SCP pattern characterized by a broader topographic distribution. Specifically, in contrast to young adults, slow negative waves at the medial frontal and right parietal regions were not distinctively enhanced in older adults. This SCP pattern implies that attentional mechanisms supporting the maintenance of working memory and inhibition networks for dual-task preparation discussed above are not efficiently employed by older individuals. A reduced involvement of such mechanisms can be explained with a deficient engagement of frontal control networks in older adults in dual tasks (Chmielewski et al., 2014), and an aging-related decrease of cognitive reserve modulated by right-hemisphere processes (Robertson, 2014). According to the present results, older adults manifested a preponderant pre-activation of visual cortical areas, pointing to a primary role of sensory analysis in dual-task preparation. Also, the positive associations detected here between the negative SCPs at left-hemisphere parietal/parieto-occipital regions and PT1/PT2 in old adults (Figure 4 and Supplementary Table 3) further imply a non-specific pre-activation of motor attention networks in the left parietal lobe (Rushworth et al., 1997, 2001, 2003; Hardwick et al., 2013) for subsequent response selection. Thus, SCP observations suggest that deficient cognitive control mechanisms limit the capacity of old subjects to prepare the two sub-tasks in advance, thus leading to prioritization of only the stimulus selection processes within the first sub-task and relying on reactive motor control (Hu et al., 2012; Kopp et al., 2014).

## Conclusion

Together, performance and SCP results demonstrate aging-related differences in pro-active preparation for dual-task processing: In young adults, the two sub-tasks of the dual-task trial can be prepared simultaneously through a rigorous attention-based pre-activation of working memory and inhibitory networks, whereas in older adults, sensory and motor attention networks appear to be non-specifically pre-activated for subsequent deferred processing of the sub-tasks under reactive cognitive control.

## Data Availability Statement

The raw data supporting the conclusions of this article will be made available by the authors, without undue reservation.

## Ethics Statement

The studies involving human participants were reviewed and approved by Ethic Committee of the Leibniz Research Centre for Working Environment and Human Factors, Dortmund, Germany. The patients/participants provided their written informed consent to participate in this study.

## Author Contributions

MF, PG, and JY designed the study. MF, SG, and PG acquired the data. JY and VK processed and analyzed the data. JY, MF, SG, RK, VK, and PG interpreted the data and wrote the manuscript text. All authors reviewed the manuscript.

## Conflict of Interest

MF was employed by the company Institut für Arbeiten Lernen Altern GmbH. The remaining authors declare that the research was conducted in the absence of any commercial or financial relationships that could be construed as a potential conflict of interest.

## Publisher’s Note

All claims expressed in this article are solely those of the authors and do not necessarily represent those of their affiliated organizations, or those of the publisher, the editors and the reviewers. Any product that may be evaluated in this article, or claim that may be made by its manufacturer, is not guaranteed or endorsed by the publisher.
