# Supplementary material for: Neural Correlates of Aging-Related Differences in Pro-active Control in a Dual Task
Source: Front Aging Neurosci. 2021 Sep 30;13:682499. doi: 10.3389/fnagi.2021.682499 (PMC8516400; doi:10.3389/fnagi.2021.682499)
Supplement: Supplementary file 1 [file Data_Sheet_1.pdf]

Supplementary Material

Supplementary Table S1. Neurocognitive scores for young and old adults.

	old	young	*t(df)*	*p*
Number of participants	118	36		
Age	70.3 (4.4)	25.2 (2.7)	$t(152) = 58.0$	0.0001
D2				
total number of correct symbols	367 (80.8)	493 (76.4)	$t(148) = 8.1$	0.0001
Digit-Symbol-Test				
total number of correct symbols	44.8 (10.4)	67.0 (10.7)	$t(152) = 11.1$	0.0001
Stroop				
word reading (sec)	14.2 (2.9)	12.7 (1.7)	$t(150) = 2.8$	0.005
color naming (sec)	21.6 (4.5)	20.5 (4.2)	$t(150) = 1.2$	ns.
interference list (sec)	43.9 (10.9)	30.9 (6.3)	$t(150) = 6.7$	0.0001
Digit span				
forward trial 1 and 2	7.4 (1.6)	8.7 (2.1)	$t(152) = 3.9$	0.0001
backward trial 1 and 2	5.7 (1.4)	8.2 (1.9)	$t(152) = 8.3$	0.0001
MWT-B				
number correct	31.5 (2.7)	27.5 (4.0)	$t(151) = 6.8$	0.0001
VLMT				
total score trials 1 to 5 (learning list A)	38.5 (9.0)	57.4 (6.4)	$t(152) = 11.9$	0.0001
Interference list (list B)	4.6 (1.6)	6.3 (1.8)	$t(151) = 5.4$	0.0001
trial 6 (list A after interference)	8.7 (2.9)	13.5 (1.5)	$t(151) = 10.8$	0.0001
trial 7 (delayed recall of list A)	8.2 (3.0)	13.3 (1.7)	$t(151) = 9.6$	0.0001
TMT*				
TMT-A (sec)	36.8 (11.1)	23.1 (6.8)	$t(142) = 7.0$	0.0001
TMT-B (sec)	94.0 (33.8)	51.0 (13.0)	$t(142) = 7.3$	0.0001
TMT B-A (sec)	57.2 (31.9)	28.5 (11.9)	$t(142) = 5.2$	0.0001

Mean values (SD) are presented unless other indicated; (sec), time to perform task; mean values of all tests indicate the number of correctly performed items; D2, test of sustained attention; VLMT, verbal learning and memory test; MWT-B, multiple choice vocabulary test; TMT, trail making test; ns., not significant; *, reduced number of participants.

Supplementary Table S2. Pearson correlation coefficient (*r*) between parameters.

(a) Young participants, N = 36.

	PT1	*PT2*	*IIT1*
Capacity	0.410 * (0.013)	-0.187 (0.274)	-0.312 (0.064)
PT1		-0.841** (0.000)	-0.621 **(0.000)
PT2			0.698 **(0.000)

(b) Old participants, N = 118.

	PT1	*PT2*	*IIT1*
Capacity	0.19 * (0.03)	0.195 (0.026)	-0.281 (0.001)
PT1		-0.857 ** (0.000)	-0.489 **(0.000)
PT2			0.444 **(0.000)

r (significance) is presented; *Capacity*, speed of processing in the dual task blocks as reflected by the mean reaction time to S1 and S2 in two SOA conditions (SOA750 and SOA0), in two blocks (B1-LC and B2-CL); *PT1*, *PT2*, preparation of T1 or T2; *IIT1*, input interference; *, significant correlation at level 0.05 (2-tailed); **, significant correlation at level 0.01 (2-tailed).

Supplementary Table S3. Regression analysis.

(a) Young participants.

Model	*R*	R^2	AdjR^2	*F*(*df*)	*p*				
PT1	0.945	0.994	0.867	33.6 (7/35)	< 0.001				
Predictors						*B*	*Beta*	*t*	*p*
PT2						-0.348	-0.753	-10.62	< 0.001
Capacity						0.005	0.181	2.69	0.02
SCP-C4						-0.74	-0.267	-3.44	0.007
SCP-Cz						0.058	0.197	2.13	0.04
SCP-P4						0.095	0.397	3.89	0.001
SCP-O2						-0.111	-0.398	-4.43	< 0.001
SCP-CP3						0.03	0192	2.4	0.02
PT2	0.938	0.881	0.869	29.5 (7/35)	< 0.001				
Predictors						*B*	*Beta*	*t*	*p*
PT1						-1.57	0.727	-7.5	< 0.001
IIT1						0.211	0.292	3.2	0.004
Capacity						0.05	0.161	1.9	0.05
SCP-C4						-0.157	-0.238	-3.07	0.005
SCP-P4						-0.1	-0.192	-2.40	0.02
SCP-F4						-0.119	-0.267	-2.65	0.01
SCP-FCz						0.128	0.223	2.37	0.02
IIT1	0.726	0.527	0.498	18.4 (2/35)	< 0.001				
Predictors						*B*	*Beta*	*t*	*p*
PT2						1.03	0.747	6.06	0.001
SCP-C4						0.388	0.439	2.31	0.03

(b) Old participants.

Model	R	R^2	AdjR^2	$F(df)$	p				
PT1	0.936	0.876	0.869	130.4 (5/117)	< 0.001				
Predictors						*B*	*Beta*	*t*	*p*
PT2						-0.3	-0.963	-27.14	< 0.001
Capacity						0.006	0.310	8.89	< 0.001
SCP-Fz						0.023	0.07	2.04	0.04
SCP-P3						0.034	0.096	2.28	0.03
SCP-PO3						0.023	0.116	2.31	0.02
PT2	0.942	0.888	0.851	176.8 (5/117)	< 0.001				
Predictors						*B*	*Beta*	*t*	*p*
PT1						-2.13	-0.845	-23.2	< 0.001
IIT1						0.017	0.34	10.41	< 0.001
Capacity						0.12	0.127	3.39	0.001
SCP-Fz						0.05	0.06	1.96	0.05
SCP-PO3						0.078	0.119	2.67	0.009
IIT1	0.611	0.373	0.351	16.8 (5/117)	< 0.001				
Predictors						*B*	*Beta*	*t*	*p*
PT2						0.531	0.525	6.84	< 0.001
Capacity						-0.018	-0.355	-4.62	< 0.001
SCP-P3						-0.129	-0.159	-1.98	0.05
SCP-O1						-0.141	-0.215	-1.97	0.05

Capacity, speed of processing in the dual task blocks as reflected by the mean reaction time to S1 and S2 in two SOA conditions (SOA750 and SOA0), in two blocks (B1-LC and B2-CL); *PT1*, *PT2*, preparation of T1 or T2; *IIT1*, input interference; *SCP*-, slow cortical potential at the respective electrode; R, R^2, AdjR^2, *B*, *Beta*, *t*, *p*, parameters of regression analysis.
